# Impact of empiric antibiotic therapy on the clinical outcome of acute calculous cholecystitis

**DOI:** 10.1007/s00423-023-03063-4

**Published:** 2023-08-29

**Authors:** Maite de Miguel-Palacio, Ana-María González-Castillo, Estela Membrilla-Fernández, María-José Pons-Fragero, Amalia Pelegrina-Manzano, Luis Grande-Posa, Ricard Morera-Casaponsa, Juan-José Sancho-Insenser

**Affiliations:** 1 Department of Surgery, Autonomous University of Barcelona, Barcelona, Spain; 2 Department of General Surgery, Hospital del Mar, Hospital del Mar Medical Research Institute (IMIM), Barcelona, Spain; 3 Department of Medicine and Life Sciences, Pompeu Fabra University, Barcelona, Spain

**Keywords:** Cholecystitis, Acute calculous cholecystitis, High-risk patients, Empiric antibiotic treatment, Tokyo Guidelines, Antibiotic adequacy

## Abstract

**Purpose:**

Although mortality and morbidity of severe acute calculous cholecystitis (ACC) are still a matter of concern, the impact of inadequate empirical antibiotic therapy has been poorly studied as a risk factor. The objective was to assess the impact of the adequacy of empirical antibiotic therapy on complication and mortality rates in ACC.

**Methods:**

This observational retrospective cohort chart-based single-center study was conducted between 2012 and 2016. A total of 963 consecutive patients were included, and pure ACC was selected. General, clinical, postoperative, and microbiological variables were collected, and risk factors and consequences of inadequate treatment were analyzed.

**Results:**

Bile, blood, and/or exudate cultures were obtained in 76.3% of patients, more often in old, male, and severely ill patients (*P* < 0.001). Patients who were cultured had a higher overall rate of postoperative complications (47.4% vs. 29.7%; *P* < 0.001), as well as of severe complications (11.6% vs. 4.7%; *P* = 0.008). Patients with positive cultures had more overall complications (54.8% vs. 39.6%; *P* = 0.001), more severe complications (16.3% vs. 6.7%; *P* = 0.001), and higher mortality rates (6% vs. 1.9%; *P* = 0.012). Patients who received inadequate empirical antibiotic therapy had a fourfold higher mortality rate than those receiving adequate therapy (*n* = 283; 12.8% vs. 3.4%; *P* = 0.003). This association was especially marked in severe ACC TG–III patients (*n* = 132; 18.2 vs. 5.1%; *P* = 0.018) and remained a predictor of mortality in a binary logistic regression (OR 4.4; 95% CI 1.3–15.3).

**Conclusion:**

Patients with positive cultures developed more complications and faced higher mortality. Adequate empirical antibiotic therapy appears to be of paramount importance in ACC, particularly in severely ill patients.

**Supplementary Information:**

The online version contains supplementary material available at 10.1007/s00423-023-03063-4.

## Introduction

Acute calculous cholecystitis (ACC) is the second most frequent cause of surgical emergency admission in the Western world. There is a general agreement on laparoscopic cholecystectomy as the preferred initial treatment of choice for patients with mild or moderate cholecystitis, as proposed by the Tokyo Guidelines 2018 (TG18) [1, 2]. For severe cases, however, there is a greater variability of therapeutic recommendations, including antibiotic therapy and/or cholecystostomy. Empiric antibiotic treatment (EAT) is central to every therapeutic option [3, 4].

As far ago as 1991, Mosdell et al. demonstrated that the adequacy of empiric antibiotic treatment is paramount to obtaining good clinical results in abdominal infections [4]. Our group updated Mosdell’s findings and demonstrated that the adequacy of antibiotic treatment in secondary peritonitis yielded a better prognosis, lowering both morbidity and mortality [5].

Regrettably, there is a lack of similar hard data about the consequences of the inadequacy of EAT on ACC. In the last decade, only a few studies have analyzed the relationship between microbiology, EAT, and clinical results in ACC. Coccolini et al. [6] and, more recently, Suh S. et al. [7] described the antibiotic resistance pattern in a small group of patients with acute cholecystitis and signaled the importance of EAT in mild or moderate acute cholecystitis. Both studies addressed the inadequacy of EAT only marginally and with some limiting methodological flaws. Moreover, these studies could not draw a clear relationship between the inadequacy of EAT and poor clinical results; however logical, this causality may seem. Most likely, the clue is to stratify the analysis by the degree of severity of the ACC. What may be irrelevant in a mild case can become a critical issue in a frail patient with ACC and severe sepsis. Recently, our group identified a concise set of major risk factors for mortality in ACC and even proposed a new score (ACME) focused on severe ACC, where the Tokyo Guidelines lack clear indications [8].

Therefore, there is a need for evidence in the ACC assessing the relevance of EAT on clinical results. Since every strategy to treat ACC includes EAT, this analysis is vital to improving clinical results.

## Material and methods

### Study type and setting

This is a retrospective single-center study performed in the Emergency Department of General Surgery of a University Hospital in Barcelona, Spain.

### Patients, inclusion, and exclusion criteria

A total of 963 consecutive patients with an initial diagnosis of acute cholecystitis between January 2012 and December 2016 were included. Data were collected by 2018, and analysis was completed by 2021.

**Fig. 1:**
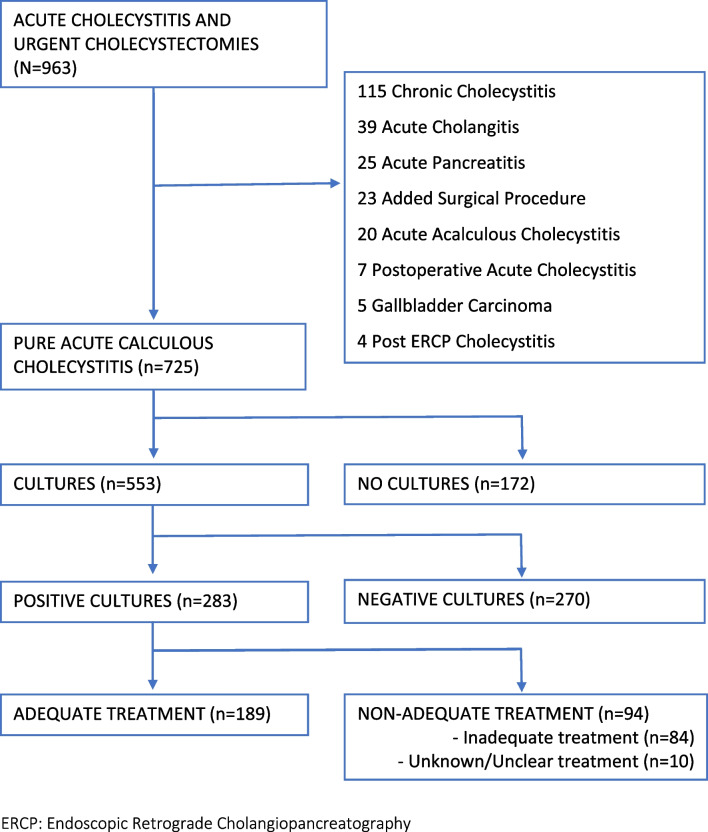
Patient selection and patient flow diagram

All patients with a clinical diagnosis of acute cholecystitis according to the Tokyo Guidelines of 2018 (TG18) were included, as well as those with a histopathologic diagnosis of ACC [9]. The study focused on patients with “pure” acute calculous cholecystitis [8]. Therefore, patients with other interrelated diagnoses, such as postoperative cholecystitis, acute cholangitis, acute pancreatitis, acalculous cholecystitis, persistent biliary colic, and cholecystitis following endoscopic retrograde cholangiopancreatography (ERCP) or gallbladder carcinoma, were excluded from the analysis (Fig. 1).

### Variables

Data were extracted and coded from the electronic medical records. A total of 211 primary variables were recorded, including demography, laboratory and imaging tests, treatments, length of stay, complications, and mortality. An additional file shows this in more detail (see Additional file 1. Variable list). Charlson’s Comorbidity Index and the ASA classification were used as preoperatory risk assessment measures. In addition, the type of initially received treatment, surgical or nonsurgical, type of empiric antibiotic administered, and duration of the antibiotic treatment (both pre-and postoperative days) were analyzed. According to the microbiological variables, genera and species of isolated germs were recorded, as well as subgroup classification according to Gram stain, oxygen requirements (aerobic/anaerobic), and morphology (cocci, bacilli, coccobacilli). The antibiotic sensitivity was determined for each isolated germ (sensitive, resistant, intermediate, or unknown), and extended-spectrum beta-lactamase bacteria (ESBLB) expression was recorded. Variables were collected in File Maker v.13 (Mountainview, CA, USA).

### Primary and secondary outcomes

The main objective of the study was to investigate the impact of inadequate EAT on mortality. This primary outcome was assessed in the first 30 days after the diagnosis of ACC or occurring at any time during the same admission. Secondary outcomes were the impact of inadequacy on the prevalence and severity of complications during the same period as mortality, as well as the description of comorbidities, use of vasopressors, and Tokyo Guidelines grade analysis.

### Adequacy estimation

Therapy was considered adequate when at least one intravenous antimicrobial was given within 24 h of admission. Antimicrobial treatment was considered adequate when used at acceptable doses and intervals, with the optimal route of administration and following local guidelines and recommendations. The adequacy of the antibiotic was determined by the in vitro susceptibility of the specific bacterial isolate, and in the case of polymicrobial infections, adequacy was determined for all isolates.

A germ was considered sensitive to the antibiotic when the minimum inhibitory concentration was less than the internationally accepted threshold [10]. When all germs isolated in a patient’s sample(s) were sensitive to the empiric antibiotics received, antibiotic therapy was considered adequate. The germs with intermediate sensitivity to the administered antibiotic were coded jointly with the resistant ones. Every treatment of a patient with ≥ 1 resistant germ or with intermediate sensitivities to the antibiotics received was labeled as inadequate.

### Interventions

The national guidelines for antibiotic treatment in intra-abdominal infection were followed, mainly using cefotaxime associated with metronidazole or piperacillin-tazobactam [11]. In patients with severe infections and risk factors for a poor outcome, empirical treatment with carbapenems was indicated, with or without association to linezolid or antifungals. Surgical patients underwent laparoscopic or open cholecystectomy depending on the surgeon’s choice. Intraoperative cultures were taken from peritoneal fluid and intravesicular bile. Veress’ needle puncture of the gallbladder was used in laparoscopic procedures, and direct needle aspiration was used in open surgery. Blood cultures were taken from both peripheral blood and/or central venous catheters. Nonsurgical patients were treated with either antibiotic therapy alone or ultrasound-guided cholecystostomy using an 8Fr catheter (SKATER ™, Argon Medical Devices, Rochester, NY, USA) by transhepatic or transperitoneal insertion.

### Statistical analysis

Univariate analysis of the association among qualitative variables was carried out using the chi-square test or Fisher’s test as needed, with results given as odds ratios with 95% confidence intervals (CIs).

The normality of the distribution of quantitative variables was estimated by the Kolmogorov‒Smirnov test and none followed a normal distribution. Therefore, these variables were summarized by their median and interquartile range (IQR), and the comparison between nonpaired means was carried out using Mann‒Whitney’s *U* test.

The multivariate analysis was completed with a forward conditional (F to enter 0.05, to remove 0.10) binary logistic regression, entering the variables with a significant association in the univariate analysis. IBM SPSS Statistics v. 25 (Rochester, MN, USA) was used in all analyses.

## Results

The median age of the study population was 69 (IQR 53–80) years old, with 23% being over 80 years old. The median Charlson Comorbidity Index score was 1.0 (from 0 to 10) points (IQR 0–2). Among the most prevalent comorbidities, a tenth of the patients suffered from chronic obstructive pulmonary disease (COPD), and 5% had a previous diagnosis of dementia [8]. An additional file shows this in more detail (see Additional file 2. Main values population).

All patients received at least one empiric intravenous antibiotic therapy dose. The overall mean duration of therapy was 6.7 days, 4 days in patients without postoperative complications, and 10 days on average in patients with complications.

### Surgery

Most of the cohort (95%) was operated on, with percutaneous cholecystostomy and/or antibiotic therapy used in a minority of the frailest patients (*n* = 13). Cholecystectomy was completed laparoscopically in 74% of patients.

### Cultures obtained

Some 553 (76%) patients had a pre- or intraoperative culture obtained from bile (*n* = 495; 68%), peritoneal fluid (*n* = 148; 20%), and/or blood (*n* = 138; 19%). Patients in whom cultures were obtained were more frequently males, older, had a higher rate of comorbidities, required more vasoactive drugs, and had a higher severity degree according to the TG18. Surgical initial treatment was chosen significantly more often in cultured patients. Those patients suffered a higher complication rate compared to those in whom cultures were not obtained, including more infectious complications, surgical site infections, and severe complications (grade > IIIa according to Clavien‒Dindo classification) [11]. Mortality was almost fourfold in the group where the cultures were taken, although statistical significance was not reached (Table 1).

**Table 1:** Differences between groups with cultures performed vs. no cultures^a^

		Global (*N* = 725)	Culture (*n* = 553)	No culture (*n* = 172)	*P*^b^	OR (95% CI)
Gender (male %)		381 (53)	309 (55.9)	72 (41.9)	0.001	1.759 (1.244–2.487)
Median age (IQR)		69 (53–80)	71 (57–80)	60 (44.25–78)	< 0.001^c^	
Charlson’s Comorbidity Index Median (IQR)		1 (0–2)	1 (0–2)	0 (0–1)	< 0.001^c^	
ACME	> 80 y/o	166 (23)	131(23.7)	35 (20.3)	0.363	1.215 (0.799–1.849)
	COPD	71 (10)	60 (10.8)	11 (6.4)	0.086	1.781 (0.914–3.471)
	Dementia	33 (5)	29 (5.2)	4 (2.3)	0.142	2.324 (0.806–6.707)
	Vasopressors	30 (4)	28 (5.1)	2 (1.2)	0.026	4.533 (1.069–19.228)
Initially surgical treatment		689 (95)	533 (96.4)	156 (90.7)	0.003	2.733 (1.383–5.402)
TG I		149 (21)	96 (17.4)	53 (30.8)	0.001^d^	1.651^d^ (1.148–2.374)
TG II		284 (39)	219 (39.6)	65 (37.8)		
TG III		292 (40)	238 (43)	54 (31.4)		
Overall complications		313 (43)	262 (47.4)	51 (29.7)	0.001	2.136 (1.470–3.084)
Infectious complications		133 (18)	114 (20.6)	19 (11)	0.005	2.091 (1.244–3.515)
SSI complications		80 (11)	71 (12.8)	9 (5.2)	0.005	2.668 (1.304–5.458)
Severe complications > IIIa^e^		74 (10)	65 (11.8)	9 (5.2)	0.014	2.412 (1.175–4.952)
Mortality		26 (3.6)	24 (4.3)	2 (1.2)	0.050	3.856 (0.902–16.486)
FTR		26/313 (8.3)	24/262 (9.2)	2/51 (3.9)	0.277	2.471 (0.565–10.798)

*OR* odds ratio, *CI* confidence interval, *IQR* interquartile range, *y/o* years old, *ACME* acute cholecystitis mortality estimation, chronic obstructive pulmonary disease, *TG* Tokyo Guidelines, *SSI* surgical site infection, *FTR* failure to rescue

^a^*N* (%)

^b^Chi-square test

^c^Mann-Whitney’s *U* test

^d^Tokyo Guidelines grade I + II vs. Tokyo Guidelines grade III

^e^According to Clavien-Dindo Classification

Germs were isolated in 283 (51%) patients in which cultures were obtained. The positivity of the culture varied depending on the sample obtained. Germs were isolated in 50% of bile cultures, 33% of blood cultures, and 30% of peritoneal fluid.

Patients in whom an extra biliary culture was obtained (peritoneal fluid cultures and/or blood cultures) presented worse results than those in whom only a bile culture sample was obtained. Accordingly, patients with extra biliary cultures experienced a greater severity according to the TG scale (TG-III 54.2% vs. 33.7%; OR, 2.33; 95% CI 1.7–3.3), higher rate of complications (63.6% vs. 33.7%; OR, 3.45; 95% CI 2.4–4.9), more severe complications (19.8% vs. 5%; OR, 4.68; 95% CI 2.6–8.6), and eight times higher mortality (8.3% vs. 1%; OR, 8.96; 95% CI 2.6–30.4).

Of the 781 samples, 420 (54%) grew identifiable germs. The bacteria most frequently isolated were *Escherichia coli* in 26% of total positive cultures (109/420 positive samples, or 37% in 107/283 patients), *Enterococcus* spp. in 16% (66/420 positive samples, or 23% in 65/283 patients), *Klebsiella* spp. in 16% (66/420 positive samples, or 22% in 62/283 patients), *Streptococcus* spp. in 11% (48/420 positive samples, or 16% in 46/283 patients), *Enterobacter* spp. in 11% (47/420 positive samples, or 16% in 44/283 patients), *Citrobacter* spp. in 4% (16/420 positive samples, or 6% in 16/283 patients), and *Clostridium* spp. in 3% (14/420 positive samples, or 5% in 14/283 patients). *Candida* spp. were isolated in 4/420 (0.9%).

### Positive cultures

As shown in Table 2, patients with positive cultures were older and had greater comorbidity. They presented a higher rate of overall complications and infectious complications compared to those in which culture(s) had been taken but no germs were isolated. Severe complications were 2.5 times higher in the culture-positive group, and mortality almost tripled in these cases (mortality 6.4% vs. 2.2%; OR, 2.99; 95% CI 1.2–7.7).

**Table 2:** Differences between groups with isolated germs vs. no germ isolation^a^

		Culture (*n* = 553)	Positive cultures (*n* = 283)	Negative cultures (*n* = 270)	*P*^b^	OR (95% CI)
Gender (male %)		309 (55.9)	160 (56.5)	149 (55.2)	0.749	1.056 (0.755–1.478)
Median age (IQR)		71 (57–80)	75 (66–82)	65.5 (49–79)	< 0.001^c^	
Charlson’s Comorbidity Index Median (IQR)		1 (0–2)	1 (0–3)	0 (0–2)	< 0.001^c^	
ACME	> 80 y/o	131(23.7)	78 (27.6)	53 (19.6)	0.028	1.558 (1.047–2.319)
	COPD	60 (10.8)	35 (12.4)	25 (9.3)	0.240	1.383 (0.804–2.380)
	Dementia	29 (5.2)	18 (6.4)	11 (4.1)	0.228	1.599 (0.741–3.452)
	Vasopressors	28 (5.1)	19 (6.7)	9 (3.3)	0.070	2.087 (0.927–6.698)
Initially surgical treatment		533 (96.4)	270 (95.4)	263 (97.4)	0.208	0.553 (0.217–1.407)
TG I		96 (1.4)	42 (14.8)	54 (20)	0.114^d^	1.313^d^ (0.937–1.840)
TG II		219 (39.6)	110 (38.9)	109 (40.4)		
TG III		238 (43)	131 (46.3)	107 (39.6)		
Overall complications		262 (47.4)	155 (54.8)	107 (39.6)	0.001	1.845 (1.316–2.587)
Infectious complications		114 (20.6)	75 (26.5)	39 (14.4)	0.001	2.136 (1.389–3.283)
SSI complications		71 (12.8)	43 (15.2)	28 (10.4)	0.090	1.549 (0.931–2.574)
Severe complications > IIIa^e^		65 (11.8)	46 (16.3)	19 (7)	0.001	2.564 (1.460–4.503)
Mortality		24 (4.3)	18 (6.4)	6 (2.2)	0.017	2.989 (1.168–7.647)
FTR		24/262 (9.2)	18/155 (11.6)	6/107 (5.6)	0.098	2.212 (0.848–5.771)

*OR* odds ratio, *CI* confidence interval, *IQR* interquartile range, *y/o* years old, *ACME* acute cholecystitis mortality estimation, *COPD* chronic obstructive pulmonary disease, *TG* Tokyo Guidelines, *SSI* surgical site infection, *FTR* failure to rescue

^a^*N* (%)

^b^Chi-square test

^c^Mann-Whitney’s *U* test

^d^Tokyo Guidelines grade I + II vs. Tokyo Guidelines grade III

^e^According to Clavien-Dindo Classification

### Impact of adequacy

In those patients with positive cultures, the adequacy or inadequacy of the empiric antibiotic treatment received was analyzed (Table 3).

**Table 3:** Differences between groups who received adequate vs. inadequate empiric treatment^a^

		Positive cultures (*n* = 283)	Adequate treatment (*n* = 189)	Non-adequate treatment (*n* = 94)	*P*^b^	OR (95% CI)
Gender (male %)		160 (56.5)	106 (56.1)	54 (57.4)	0.828	0.946 (0.574–1.559)
Median age (IQR)		75 (66–82)	72 (63.5–80)	78 (71.75–85)	0.001^c^	
Charlson’s Comorbidity Index Median (IQR)		1 (0–3)	1 (0–2)	1 (0–3)	0.209^c^	
ACME	> 80 y/o	78 (27.6)	45 (23.8)	33 (35.1)	0.045	0.578 (0.337–0.991)
	COPD	35 (12.4)	19 (10.1)	16 (17)	0.094	0.545 (0.266–1.116)
	Dementia	18 (6.4)	10 (5.3)	8 (8.5)	0.296	0.601 (0.229–1.576)
	Vasopressors	19 (6.7)	13 (6.9)	6 (6.4)	0.875	1.083 (0.398–2.947)
Initially surgical treatment		270 (95.4)	181 (95.8)	89 (94.7)	0.681	1.271 (0.404–3.997)
TG I		42 (14.8)	26 (13.8)	16 (17)	0.618^d^	1.254^d^ (0.761–2.064)
TG II		110 (38.9)	72 (38.1)	38 (40.4)		
TG III		131 (46.3)	91 (48.1)	40 (42.6)		
Overall complications		155 (54.8)	98 (51.9)	57 (60.6)	0.162	0.699 (0.423–1.156)
Infectious complications		75 (26.5)	46 (24.3)	29 (30.9)	0.242	0.721 (0.416–1.249)
SSI complications		43 (15.2)	24 (12.7)	19 (20.2)	0.097	0.574 (0.296–1.112)
Severe complications > IIIa^e^		46 (16.3)	27 (14.3)	19 (20.2)	0.203	0.658 (0.344–1.257)
Mortality		18 (6.4)	6 (3.2)	12 (12.8)	0.002	0.224 (0.081–0.618)
FTR		18/155 (11.6)	6/98 (6.1)	12/57 (21.1)	0.005	0.245 (0.086–0.694)

*OR* odds ratio, *CI* confidence interval, *IQR* interquartile range, y/o: years old, *ACME* acute cholecystitis mortality estimation, *COPD* chronic obstructive pulmonary disease, *TG* Tokyo Guidelines, *SSI* surgical site infection, *FTR* failure to rescue

^a^*N* (%)

^b^Chi-square test

^c^Mann-Whitney’s *U* test

^d^Tokyo Guidelines grade I + II vs. Tokyo Guidelines grade III

^e^According to Clavien-Dindo Classification

Gram-negative bacilli from the *Enterobacteriaceae* family were the germs most frequently involved in inadequacy (*n* = 55), especially those producing extended-spectrum beta-lactamases (ESBLs), which were found in 36 patients (12.7% of patients with positive cultures). They were mainly *E*. *coli*, *Enterobacter* spp., and *Citrobacter* spp. The second cluster in the frequency of inadequacy was *Enterococcus* spp. involved in one third of cases (*n* = 30).

Patients with adequate EAT had a considerably lower mortality rate than those who received inadequate treatment (3.2% vs. 12.8%; OR, 0.22; 95% CI 0.1–0.6), both in cases of mild ACC-moderate (mortality 2% vs. 7.4%; OR, 0.26; 95% CI 0.05–1.5) and more strikingly in severe TG-III cases of ACC (mortality 4.4% vs. 20%; OR, 0.18; 95% CI 0.1–0.7). As shown in Table 3, global complications, severe complications (Clavien-Dindo > IIIa), infectious complications, and surgical site infections were more frequent in patients with inadequate EAT, although statistical significance was not reached.

Nonadequacy was an independent variable predicting mortality in a binary logistic regression model that also included the four variables of ACME [8] with a significant association with mortality in the univariate analysis: age over 80 years old, the requirement of vasopressor therapy, and the preoperative diagnosis of dementia and/or chronic obstructive pulmonary disease (COPD). Thus, as shown in Table 4, patients with inadequate EAT were 4.4 times more likely to die than those with adequate EAT (OR, 4.4; 95% CI 1.3–15.3).

**Table 4:** Binary logistic regression on mortality between adequate vs. non-adequate EAT groups

	*B* (SE)	*P*	OR	95% CI
EAT adequacy^a^	1.479 (0.638)	0.020	4.391	1.258–15.322
Age > 80 y/o	1.867 (0.644)	0.004	6.466	1.830–22.855
Vasopressors use	2.372 (0.758)	0.002	10.716	2.426–47.323
Dementia	1.693 (0.860)	0.049	5.436	1.008–29.316
COPD	1.707 (0.673)	0.011	5.512	1.475–20.603

*B(SE)* coefficient for the constant (standard error), *OR* odds ratio, *CI* confidence interval, *EAT* empirical antibiotic therapy, *y/o* years old, *COPD* chronic obstructive pulmonary disease

^a^Inadequate or unknown/unclear treatment *vs.* adequate treatment

## Discussion

The current clinical study investigates the impact of not prescribing adequate empirical therapy to patients treated for acute cholecystitis. Most of the cohort (95%) was operated on, with percutaneous cholecystostomy and/or antibiotic therapy used in a minority of the frailest patients (*n* = 13).

### Selection of the patient and the sample for culture and determination of adequacy

As stated in the TG18, in our department, it was not compulsory to culture every sample (bile, blood and peritoneal fluid) from every patient [3]. However, surgical teams tended both to obtain cultures more often from severe patients and to obtain blood and peritoneal fluid cultures (in addition to bile cultures) from the frailest patients. This obvious observational *bias* guided our subgroup analysis, assessing the effect of inadequacy in mild/moderate or severe groups of ACC separately.

Although there is no international standard to define EAT adequacy, we used the most widely employed definition of EAT adequacy in the English scientific literature [12, 13].

### Empirical antibiotic treatment of acute cholecystitis

In recent years, few studies have been published on the microbiology of acute calculous cholecystitis and empirical antibiotic therapy. In a registry-based (CIAO and CIAOW) multicentric observational study, the group of Coccolini et al. accurately described the antibiotic resistance pattern in an acute cholecystitis subpopulation (*n* = 567). They also found a significant association between mortality and inadequacy in the univariate analysis. Their population was decidedly heterogeneous, and nevertheless, their main conclusion agrees with the current study, although inadequacy was not identified as an independent factor in their multivariate analysis [6].

Suh et al. [7] described the trends in microbial etiology and antibiotic resistance over the years focusing solely on bile cultures and the importance of the adequacy of antibiotic therapy in a selected and well-analyzed population. Unfortunately, unlike the current study, their group included only mild and moderate acute cholecystitis, excluding patients with severe acute cholecystitis (TG18 grade III). In addition, patients with acalculous cholecystitis or with concurrent pancreatitis were included, increasing heterogeneity. They could not draw a relationship between inappropriate empirical antibiotics and clinical results, probably because severe cases were not included. Despite these limitations and coinciding with our results, they conclude that surgical control of the infectious focus is essential.

In PEANUTS II, a multicenter randomized clinical trial of noninferiority, clinical results were worse in the group without preoperative antibiotics compared with the group who did receive an empiric prophylactic antibiotic in urgent cholecystectomies for uncomplicated mild or moderate ACC [14]. They observed a higher complication rate, especially infectious complications. Thus, although surgery plays a key role in the control of the infectious focus in mild and moderate phases, we cannot disregard the central role of antibiotics, even in these early stages of the disease.

To our knowledge, there is a notorious lack of relevant information on the role of antibiotic adequacy in severe acute cholecystitis. In the current study, we wanted to fill this gap, including in the analysis the most severe patients, since they bear the greatest margin for improvement both in mortality and morbidity.

On the other hand, in recent years, frailty has acquired indisputable relevance when making therapeutic decisions in elderly patients. Several frailty scales have been described to classify patients trying to facilitate the surgeon’s decision in selecting which patients, despite their advanced age, could benefit from surgical treatment. In this subgroup of elderly patients, perhaps less likely to receive a final surgical treatment, the role of antibiotic therapy appears essential to achieve control of the infectious focus [15–17].

### Multiresistant bacteria

The worrisome growth rate of multidrug-resistant bacteria in our environment is well known. Other groups have already described the presence of ESBLB in patients with acute cholecystitis, and the current study confirms these findings. The presence of *Enterococcus* spp. is also noteworthy in a third of the patients who did not receive adequate initial EAT [6, 7, 18–21].

### Clinical guidelines

The Spanish guidelines with recommendations for intra-abdominal infection [22] and the revised Guidelines of the Surgical Infection Society [23] emphasize limiting the duration of antibiotic treatment but fail to offer differential therapeutic options for severe ACC patients.

It is possible that if the EAT protocols are modified to empirically include broad-spectrum antibiotics in unselected patients, we will witness a surge of new antibiotic resistance. However, we believe that by widening the spectrum in severe patients, morbidity and mortality will improve. On the other hand, the duration of EAT could be drastically reduced, following the recommendations for short-term therapy and de-escalating treatments as soon as germ isolation is confirmed [24].

### Limitations of the study

The current study presents the limitations of retrospective cohort studies, such as the possible selection bias of cases, since it is a single-center study that covers a specific population of the city of Barcelona, and the lack of data on some variables. However, the data were collected and reviewed by two authors, which reduces the risk of misclassification, increases reliability and reduces interobserver differences. The decision to obtain cultures and from where the samples were taken was entirely left to the surgical team’s preference. The results imply, however, that the patient and site selection to obtain cultures were not random, as cultured patients had worse clinical results, suggesting a positive selection bias.

## Conclusion

There was a greater tendency to perform cultures in older and male patients, with more severe ACC. Patients with positive cultures developed more complications and higher mortality than patients without germ isolation. Non-adequate empirical antibiotic therapy was associated with a higher mortality rate, especially in severe ACC TG18 grade III.

Therefore, adequate empirical antibiotic therapy appears to be of paramount importance in ACC, and those patients with severe ACC will probably benefit from a wider empirical broad-spectrum antibiotic therapy securing coverage of ESBL and *Enterococcus* spp.

### Supplementary Information

Below is the link to the electronic supplementary material.

Supplementary file1 (PDF 543 KB)

Supplementary file2 (PDF 566 KB)

## Data Availability

The datasets used and/or analyzed during the current study are available from the corresponding author on reasonable request.
