# Supplementary material for: Impact of empiric antibiotic therapy on the clinical outcome of acute calculous cholecystitis
Source: Langenbecks Arch Surg. 2023 Aug 29;408(1):345. doi: 10.1007/s00423-023-03063-4 (PMC10465626; doi:10.1007/s00423-023-03063-4)
Supplement: Supplementary file 1 — Supplementary file1 (PDF 543 KB) [file 423_2023_3063_MOESM1_ESM.pdf]

**DEMOGRAPHICS**	AGE			
		Age >80 (Y/N)		
	GENDER			
	ASA			
	WHEIGHT			
	BMI			
	REGISTRATION NUMBER			
	REGISTRATION DATE			
	UNDERLYING DISEASE (Y/N)			
	CHARLSON	Prior Myocardial Infarction		
		Congestive Heart Failure		
		Peripheral Vascular Disease		
		Cerebrovascular Disease		
		Dementia		
		Chronic Pulmonary Disease		
		Rheumatologic Disease		
		Peptic Ulcer Disease		
		Mild Liver Disease		
		Diabetes		
		Cerebrovascular (hemiplegia) Event		
		Moderate-to-severe Renal Disease		
		Diabetes with Chronic Complications		
		Cancer Without Metastases		
		Leukemia		
		Lymphoma		
		Moderate or Severe Liver Disease		
		Metastatic Solid Tumor		
		Acquired Immuno-Deficiency Syndrome (AIDS)		
	CHARLSON SCORE			
	OBSERVATIONS			
**PREOPERATORY**	EMERGENCY CONSULTATION	Date of Emergency Consultation		
		Previous Symptomatic Cholelithiasis		
		Number of Previous Emergency Consultations		
		Hospital Readmission (Y/N)		
		Preop Axillary Temperature		
		Preop FiO2		
		Onset of Pain		
		Preop Pain >72 hours		
		Preop Palpable Mass		
		Preop Murphy Sign		
		Preop Tachypnea		
		Systemic Inflammatory Response Syndrome		
	PREOPERATORY BLOOD TESTS	Preop Lactic Acid		
		Preop CRP		
		Preop Creatinine		
		Preop Amylase		
		Preop Lipase		
		Preop Bilirubin		
		Preop Gamma-GT		
		Preop GOT		
		Preop GPT		
		Preop Alkaline Phosphatase		
		Preop Leukocytes		
		PreopP Arterial Blood Gases (Y/N)		
		Preop PaO2		
		Preop PC02		
		Preop INR		
		Preop Platelets		
	IMAGING TEST	Ultrasonography (Y/N)		
		TC Scan (Y/N)		
		Preop Emphysematous		
		Preop Liver Abscess		
		Preop Gangrenous		
		Preop Bile Peritonitis		
	DIAGNOSIS	Complete Diagnosis		
		"Pure" Classification (Y/N)		
		Other Concomitant Diagnosis		
**TOKYO GUIDELINES**	GRADE	TG Grade I		
		TG Grade II		
		TG Grade III		

		TG Vasopressors		
		TG Consciousness		
		TG Renal Failure		
		TG INR 1.5		
		TG Leukocytes		
		TG Marked Inflammatory		
		TG Oliguria		
		TG PAFI 300		
		TG PAFI UK		
		TG Platelets 100K		
**TREATMENT**	INITIAL TREATMENT	Medical treatment (Y/N)		
		Surgery (Y/N)		
		Cholecystostomy (Y/N)		
		Final Treatment		
	SURGERY	Final Surgical Treatment (Y/N)		
		Surgery Start Time		
		Surgery End Time		
		Surgery Duration		
		Surgeon		
		First Assistant		
		Second Assistant		
		Years Active Surgeon		
		Operative Summary		
		Cholangiography (Y/N)		
		Surgical Approach	Open/Lap	
			Converted (Y/N)	
		Associated Surgical Gestures (Y/N)		
			Number of Gestures	
		Intraop Type of Cholecistitis	Phegmonous	
			Gangrenous	
			Perforated	
	ANTIBIOTICS	Antibiotics 1		
		Antibiotics 2		
		Antibiotics 3		
		Duration of Treatment		
		Duration of Treatment After Surgery		
		Antibiotics preop		
		Antibiotics postop		
		Observations		
		Medical Treatment Results		
	PATHOLOGY	Acute Cholecystitis (Y/N)		
		Details		
**COMPLICATIONS**	BÜCHLER COMPLICATIONS	Büchler Score		
		Abscess		
		Cholangitis or Cholecystitis		
		Surgical Site Infection		
		Cerebrovascular Complication		
		>72h Abdominal Pain		
		Exitus		
		>72h Fever		
		Biliary Fistula		
		Hemorrhage		
		Acute Myocardial Infarction		
		Jaundice		
		Renal Failure		
		Pancreatitits		
		Peritonitis		
		Pneumonia		
		Reoperation		
		>72h Sepsis		
		Septic Shock		
		Pulmonary Embolism		
		Thrombosis		
	COMPLICATIONS CLAVIEN-DINDO	Complications (Y/N)		
		Grade		
		Severe Complications >3a		
	EXITUS (Y/N)			
		Cardiac Cause		
		Cholecystitis Cause		
		Undetermined Cause		

		Infectious Cause		
		Neoplasic Cause		
		Respiratory Cause		
		Exitus Day		
	ADMISSION DATE			
	DISCHARGE DATE			
	LEGTH OF STAY			
	DISCHARGE FORM			
	LAST FOLLOW UP			
	POSTOPERATIVE LENGTH OF STAY			
	INFECTIOUS COMPLICATIONS			
		Surgical Site Infection		
	COMPLICATION DETAILS			
	READMISSION (Y/N)			
	REOPERATION (Y/N)			
		Number of Reoperations		
**MICROBIOLOGY**	CULTURE (Y/N)			
		Bile Culture (Y/N)		
			Gram	
			Germs	
		Extrabiliary Culture (Y/N)		
			Peritoneal Fluid Culture (Y/N)	
				Gram
				Germs
			Blood Culture (Y/N)	
				Gram
				Germs
				Only Blood Growing
		Isolated Germs (Y/N)		
			Germ1	
			Germ2	
			Germ3	
			Germ4	
			Germ5	
			Bacteria (Y/N)	
				Gram
				Group
				Genus
				Species
				Aerobic
				Anaerobic
				Count *Bacteroides spp*
				Count *Clostridium spp*
				Count *Enterobacter spp*
				Count *Enterococcus spp*
				Count *Escherichia coli*
				Count *Klebsiella spp*
				Count *Pseudomonas spp*
				Count *Staphylococcus sp*
				Count *Streptococcus spp*
				Count ESBL
			Fungal (Y/N)	
				Count Candida
			Sensitivity (Y/N/UKUC)	
				Amikacin
				Amoxi/clav
				Ampicillin
				Aztreonam
				Cefazolin
				Cefotaxime
				Ciprofloxacin
				Clindamicin
				Ertapenem
				Gentamicin
				Imipenem
				Levofloxacin
				Linezolid
				Meropenem
				Piperacillin/tazobactam
				Tobramicin

				Vancomicin
