# Supplementary material for: Impact of empiric antibiotic therapy on the clinical outcome of acute calculous cholecystitis
Source: Langenbecks Arch Surg. 2023 Aug 29;408(1):345. doi: 10.1007/s00423-023-03063-4 (PMC10465626; doi:10.1007/s00423-023-03063-4)
Supplement: Supplementary file 2 — Supplementary file2 (PDF 566 KB) [file 423_2023_3063_MOESM2_ESM.pdf]

VARIABLES						N (%)	N	Median	Min	Max	IQR
**DEMOGRAPHICS**	AGE						725	69	18	96	53-80
		Age ≥80 (Y/N)				25.9	188/725				
	GENDER (male)					52.6	381/725				
	ASA						701	2	1	4	2-3
	WEIGHT						405	78	42	145	69-88
	BMI						234	28.7	17.8	51	25.7-33.1
	UNDERLYING DISEASE (Y/N)					51.2	371/725				
	CHARLSON	Prior Myocardial Infarction				8.3	60/725				
		Congestive Heart Failure				14.6	106/725				
		Peripheral Vascular Disease				9	65/725				
		Cerebrovascular Disease				7.4	54/725				
		Dementia				4.6	33/725				
		Chronic Pulmonary Disease				9.8	71/725				
		Rheumatologic Disease				1.2	9/725				
		Peptic Ulcer Disease				3.4	25/725				
		Mild Liver Disease				3.3	24/725				
		Diabetes				22.1	160/725				
		Cerebrovascular (hemiplegia) Event				0.6	4/725				
		Moderate-to-severe Renal Disease				8.7	63/725				
		Diabetes with Chronic Complications				3.3	24/725				
		Cancer Without Metastases				4.3	31/725				
		Leukemia				0.3	2/725				
		Lymphoma				0.6	4/725				
		Moderate or Severe Liver Disease				2.3	17/725				
		Metastatic Solid Tumor				1.2	9/725				
		Acquired Immuno-Deficiency Syndrome (AIDS)				0	0/725				
	CHARLSON SCORE						725	1	0	10	0-2
**PREOPERATORY**	EMERGENCY CONSULTATION	Previous Symptomatic Cholelithiasis				25	181/725				
		Number of Previous Emergency Consultations					181	1	1	6	1-2
		Preop Axillary Temperature					578	36.3	34.4	38.3	36-37.2
		Onset of Pain					725	2	0	365	1-4
		Preop Pain >72 hours				37.1	269/725				
		Preop Palpable Mass				23.7	170/717				
		Preop Murphy Sign				47.6	269/565				
		Preop Tachypnea				10	69/691				
		Systemic Inflammatory Response Syndrome				34.7	227/654				
	PREOPERATORY BLOOD TESTS	Preop Lactic Acid (mmoL/L)					351	1.5	0.5	4.5	1.1-2.2
		Preop CRP (mg/dL)					662	12	0	66.4	2.6-25
		Preop Creatinine (mg/dL)					720	0.82	0.20	8.58	0.65-1.1
		Preop Amylase					5	53	22	55	29-54.5
		Preop Lipase					677	27	0.02	9000	20-40
		Preop Bilirubin					712	0.88	0.09	18.53	0.52-1.64
		Preop Gamma-GT					41	118	9	1572	69-227
		Preop GOT					720	27	8	3663	18-62
		Preop GPT					705	29	5	1830	17-72
		Preop Alkaline Phosphatase					653	94	0.6	677	70-145
		Preop Leukocytes					722	13760	390	42660	[illegible]0880-17710
		Preop INR					718	1.2	0.68	2.96	1.07-1.37
		Preop Platelets					721	222000	31000	618000	[illegible]000-286000
	IMAGING TEST	Ultrasonography confirms diagnosis(Y/N)				91.5	658/719				
		TC Scan (Y/N)				12.5	90/719				
		Preop Emphysematous				0.6	4/719				
		Preop Liver Abscess				3.3	24/719				
		Preop Gangrenous				7.5	54/719				
		Preop Bile Peritonitis				3.1	22/719				
**TOKYO GUIDELINES**	GRADE	TG Grade I				20.7	150/725				
		TG Grade II				40.1	291/725				
		TG Grade III				39.2	284/725				
		TG Vasopressors				4.1	30/725				
		TG Consciousness				20.1	146/725				
		TG Renal Failure				9.6	69/720				
		TG INR 1.5				22.9	23/718				
		TG Leukocytes				23	166/722				
		TG Marked Inflammatory				11.9	86/725				
		TG Oliguria				6.1	44/725				
		TG PAFI 300				2.6	19/725				
		TG Platelets 100K				3.2	23/721				
**TREATMENT**	INITIAL TREATMENT	Medical treatment				3.3	24/725				
		Surgery				95	689/725				
		Cholecystostomy				1.7	12/725				
	SURGERY	Final Surgical Treatment				95.7	694/725				
		Surgery Duration (min)					684	145	30	524	108-184
		Cholangiography				21.9	152/694				
		Surgical Approach	Laparoscopic			90.1	625/694				
			Converted			14.9	93/625				
		Associated Surgical Gestures				23.5	163/694				
	EMPIRIC ANTIBIOTICS	Amoxicillin/Clavulanic Acid				17.9	130/725				
		Aztreonam / Metronidazole				5.7	41/725				
		Cefotaxime +/- Metronidazole				46.6	338/725				
		Ceftriaxone +/- Metronidazole				1.4	10/725				
		Ciprofloxacin +/- Clindamycin				1.1	8/725				
		Ciprofloxacin / Metronidazole				1.2	9/725				
		Carbapenem +/- Linezolid				2.6	19/725				
		Piperacillin / Tazobactam				21.9	159/725				
		Other				1.5	11/725				
	PATHOLOGY	Acute Cholecystitis (Y/N)				91.7	613/669				
**COMPLICATIONS**	BÜCHLER COMPLICATIONS	Büchler Score					725	3	0	95	3-7
		Abscess				6.9	50/725				
		Surgical Site Infection				6.3	46/725				
		Cerebrovascular Complication				0.1	1/725				
		>72h Abdominal Pain				21.4	155/725				
		Exitus				3.6	26/725				
		>72h Fever				2.9	21/725				
		Biliary Fistula				3.2	23/725				
		Hemorrhage				3	22/725				
		Acute Myocardial Infarction				0.8	6/725				
		Jaundice				14.2	103/725				
		Renal Failure				10.6	77/725				
		Pancreatitits				1.8	13/725				
		Peritonitis				7	51/725				

		Pneumonia				3.3	24/725				
		Reoperation				3.2	23/725				
		>72h Sepsis				4.6	33/725				
		Septic Shock				6.6	48/725				
		Pulmonary Embolism				0.1	1/725				
		Thrombosis				0	0/725				
	COMPLICATIONS CLAVIEN-DINDO	Complications (Y/N)				43.2	313/725				
		Compliation grade	Grade I			33.5	105/313				
			Grade II			28.4	89/313				
			Grade III			19.8	62/313				
			Grade IV			9.9	31/313				
			Grade V			8.3	26/313				
		Severe Complications >3a				23.6	74/313				
	EXITUS					3.6	26/725				
	LEGTH OF STAY						725	4	0	103	2-7
	INFECTIOUS COMPLICATIONS					42.5	133/313				
		Surgical Site Infection				25.6	80/313				
	READMISSION (Y/N)					0.4	3/725				
	REOPERATION (Y/N)					1.8	13/725				
		Number of Reoperations					1	1	1	1	1-1
**MICROBIOLOGY**	CULTURE (Y/N)					76.3	553/725				
		Bile Culture				68.3	495/725				
			Positive Cultures			50.3	249/495				
			Gram	Gram positive		43.8	109/249				
				Gram negative		76.3	190/249				
		Extrabiliary Culture				34.9	253/725				
			Peritoneal Fluid Culture			20.4	148/725				
				Positive cultures		29.7	44/148				
				Gram positive		50	22/44				
				Gram negative		75	33/44				
			Blood Culture			19	138/725				
				Positive cultures		32.6	45/138				
				Gram positive		40	18/45				
				Gram negative		80	36/45				
				Only Blood Growth		7.2	10/138				
		Global Positive Cultures				51.2	283/553				
			Bacterial			99.3	281/283				
				Aerobic		97.5	274/281				
				Anaerobic		7.5	21/281				
				Count *Escherichia coli*		38.1	107/281				
				Count *Enterococcus spp*		22.8	64/281				
				Count *Klebsiella spp*		22.1	62/281				
				Count *Streptococcus spp*		16.4	46/281				
				Count *Enterobacter spp*		15.7	44/281				
				Count *Citrobacter spp*		5.7	16/281				
				Count *Clostridium spp*		5	14/281				
				Count *Staphylococcus spp*		3.6	10/281				
				Count *Morganella spp*		1.8	5/281				
				Count *Bacteroides spp*		1.8	5/281				
				Count *Haemophilus spp*		1.4	4/281				
				Count *Proteus spp*		1.4	4/281				
				Count *Aeromonas spp*		1.1	3/281				
				Count *Pseudomonas spp*		1.1	3/281				
				Count *Hafnia spp*		1.1	3/281				
				Count *Lactobacillus spp*		0.7	2/281				
				Count *Serratia spp*		0.7	2/281				
				Count *Corynebacterium spp*		0.7	2/281				
				Count *Acinetobacter spp*		0.4	1/281				
				Count *Actinomyces spp*		0.4	1/281				
				Count *Campylobacter spp*		0.4	1/281				
				Count *Cronobacter spp*		0.4	1/281				
				Count *Gemella spp*		0.4	1/281				
				Count *Lactococcus spp*		0.4	1/281				
				Count *Providencia spp*		0.4	1/281				
				Count ESBL		14.2	40/281				
			Fungal			1.4	4/283				
				Count *Candida* spp		100	4/4				
